# Administration of the GABA_A _receptor antagonist picrotoxin into rat supramammillary nucleus induces c-Fos in reward-related brain structures. Supramammillary picrotoxin and c-Fos expression

**DOI:** 10.1186/1471-2202-11-101

**Published:** 2010-08-17

**Authors:** Rick Shin, Satoshi Ikemoto

**Affiliations:** 1 Behavioral Neuroscience Branch, National Institute on Drug Abuse, National Institutes of Health, Department of Health and Human Services, Baltimore, Maryland 21224, USA

## Abstract

**Background:**

Picrotoxin blocks GABA_A _receptors, whose activation typically inhibits neuronal firing activity. We recently found that rats learn to selectively self-administer picrotoxin or bicuculline, another GABA_A _receptor antagonist, into the supramammillary nucleus (SuM), a posterior hypothalamic structure localized anterior to the ventral tegmental area. Other drugs such as nicotine or the excitatory amino acid AMPA are also self-administered into the SuM. The SuM appears to be functionally linked with the mesolimbic dopamine system and is closely connected with other brain structures that are implicated in motivational processes, including the prefrontal cortex, septal area, preoptic area, lateral hypothalamic area and dorsal raphe nucleus. Here, we hypothesized that these brain structures are activated by picrotoxin injections into the SuM.

**Results:**

Picrotoxin administration into the SuM markedly facilitated locomotion and rearing. Further, it increased c-Fos expression in this region, suggesting blockade of tonic inhibition and thus the disinhibition of local neurons. This manipulation also increased c-Fos expression in structures including the ventral tegmental area, medial shell of the nucleus accumbens, medial prefrontal cortex, septal area, preoptic area, lateral hypothalamic area and dorsal raphe nucleus.

**Conclusions:**

Picrotoxin administration into the SuM appears to disinhibit local neurons and recruits activation of brain structures associated with motivational processes, including the mesolimbic dopamine system, prefrontal cortex, septal area, preoptic area, lateral hypothalamic area and dorsal raphe nucleus. These regions may be involved in mediating positive motivational effects triggered by intra-SuM picrotoxin.

## Background

Recent intracranial self-administration studies have helped to define key brain structures involved in positive motivational processes involved in approach/seeking [1]. One such structure is the supramammillary nucleus (SuM), located in the posterior hypothalamic area, just dorsal to the mammillary body and anterior to the ventral tegmental area (VTA). The SuM was initially implicated in reward-related processes by the finding that rats learn instrumental responses to obtain brief electrical stimulation in the vicinity of the SuM [2]. We recently found that rats readily learn to lever-press for infusions of GABA_A _receptor antagonists, picrotoxin or bicuculline [3], the excitatory amino acid AMPA [4], or nicotine [5] into the SuM, suggesting that activation of supramammillary neurons recruits approach-related motivational processes [1].

We have also shown low systemic doses of dopamine receptor antagonists decrease rats' self-administration of picrotoxin or AMPA into the SuM [3,4]. These findings suggest that motivational effects of supramammillary manipulations depend on intact dopamine transmission. In addition, AMPA administration into the SuM increases extracellular dopamine concentrations in the ventral striatum as measured by microdialysis [4]. This result suggests that activation of supramammillary neurons appears to recruit activation of the mesolimbic dopamine system.

Conversely, some manipulations that activate VTA dopamine neurons appear to activate supramammillary neurons. One manipulation that supports robust self-administration is the cholinergic receptor agonist carbachol into the posterior VTA [6], which increases extracellular dopamine in the ventral striatum [7], suggesting that this manipulation activates the mesolimbic dopamine system. The administration of carbachol into the posterior VTA was found to robustly increase the transcription factor c-Fos in the SuM [8]. SuM c-Fos counts were positively correlated with locomotor counts increased by carbachol administration into the VTA, suggesting that SuM neurons participate in motivational effects triggered by posterior VTA activation. These findings suggest that supramammillary neurons interact with the mesolimbic dopamine system.

Although the SuM appears to be functionally linked with the dopaminergic projection from the VTA to the ventral striatum, connectivity between the SuM and these regions is either absent or scarce [9-14]. Additional connectivity analysis suggests that several regions that are reciprocally connected with the SuM are also reciprocally connected with the VTA [1]. These include the medial prefrontal cortex, septal area, preoptic area, lateral hypothalamic area and dorsal raphe nucleus. Moreover, these efferent and afferent regions of the SuM and VTA appear to be closely interlinked. Because these regions have also been implicated in reward-related processes, these brain regions, the mesolimbic dopamine system and SuM may constitute a network module that mediates approach-related motivational processes [1].

Using c-Fos as a marker for neuronal activation, we sought to address the following hypotheses: 1) the administration of GABA_A _receptor antagonist picrotoxin into the SuM activates its' local neurons, because the GABA_A _receptor antagonist blocks tonic inhibition, leading to neuronal disinhibition; 2) picrotoxin administration into the SuM activates the mesolimbic dopamine system; 3) picrotoxin administration into the SuM activates the brain regions that are involved in motivational processes and reciprocally connected with both SuM and VTA: medial prefrontal cortex, septum, preoptic area, lateral hypothalamic area, and dorsal raphe nucleus.

In addition, we examined whether intra-SuM administration of picrotoxin facilitates locomotor activity. Positive motivational manipulations such as brain stimulation reward and drugs of abuse are known to acutely facilitate locomotor activity in rats [15,16]. Because our previous studies show positive motivational effects of intra-SuM picrotoxin, we hypothesized that this manipulation will facilitate locomotor activity in open fields. The present study focused on effects of acute intra-SuM picrotoxin administration on locomotor activity and c-Fos expressions in reward-related brain structures. Therefore, the study emphasized on initial unconditioned processes, rather than conditioned processes, triggered by SuM manipulations.

## Results and Discussion

**Figure 1:**
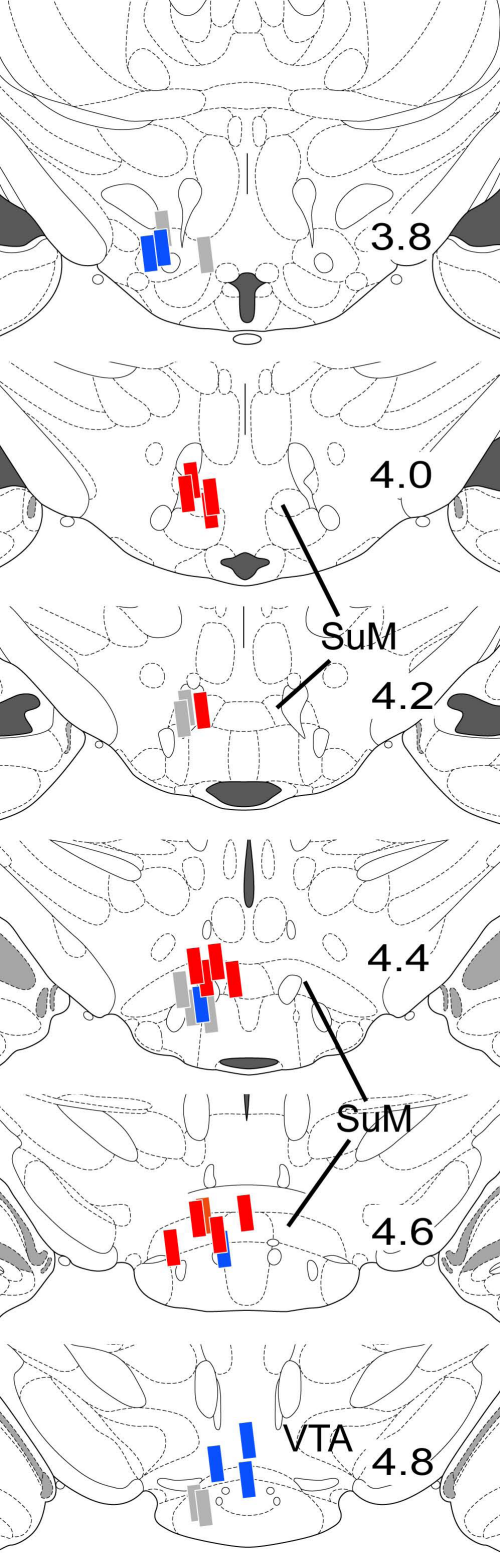
**Locations of the tips of injection cannulae**. Injection sites found within the supramammillary nucleus are marked by red rectangles (N = 14). Blue rectangles indicate injection sites outside the supramammillary nucleus (N = 7) and gray indicate vehicle injection sites (N = 9).

Each rat unilaterally received picrotoxin or vehicle into the vicinity of the SuM through a permanent guide cannula. After a locomotor activity test, each rat was killed for histological processes of c-Fos expression and cannula placement. Each rat's cannula placement was verified with microscopic examination and shown in Figure 1.

### Effects of SuM picrotoxin administration on locomotor activity

**Figure 2:**
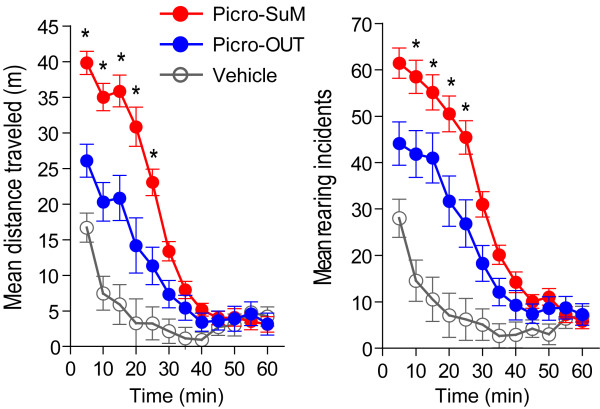
**Locomotor and rearing counts in open-field chambers after microinjections of picrotoxin or vehicle into the vicinity of the supramammillary nucleus**. Left and right panels show mean locomotion and rearing, respectively, as a function of injection manipulation group and time. * *P *< 0.05, significantly greater than respective vehicle value. Abbreviations: Picro, picrotoxin; OUT, outside of the SuM.

Unilateral administration of picrotoxin (0.3 mM in 0.3 μl) into the SuM immediately facilitated locomotion and rearing that then gradually decreased over the next 40 min (Figure 2). Because rats that received artificial cerebrospinal fluid into regions just outside the SuM showed similar locomotor/rearing counts as those receiving it into the SuM, the data of both groups were combined together as vehicle control data. During the 60 min observation period, the rats that received picrotoxin into the SuM traveled and reared approximately 4 times more than those that received vehicle. These observations were verified by mixed 3 × 12 (injection manipulation group × 5-min block) ANOVA/MANOVAs. We found significant group × block interactions, *F*_22,34 _= 2.72, *P *= 0.0042 for locomotion and *F*_22,34 _= 2.31, *P *= 0.013 for rearing. These results suggest that picrotoxin injections into the SuM markedly facilitate locomotion and rearing. Because the SuM is closely connected with visceral and affective processing structures rather than motor processing structures and because previous studies suggest positive motivational role of this structure, it is tempting to suggest that locomotor activity triggered by intra-SuM picrotoxin is secondarily facilitated through motivational processes, thereby reflecting unconditioned motivational processes of the manipulation.

### Effects of SuM picrotoxin administration on c-Fos in reward related structures

**Figure 3:**
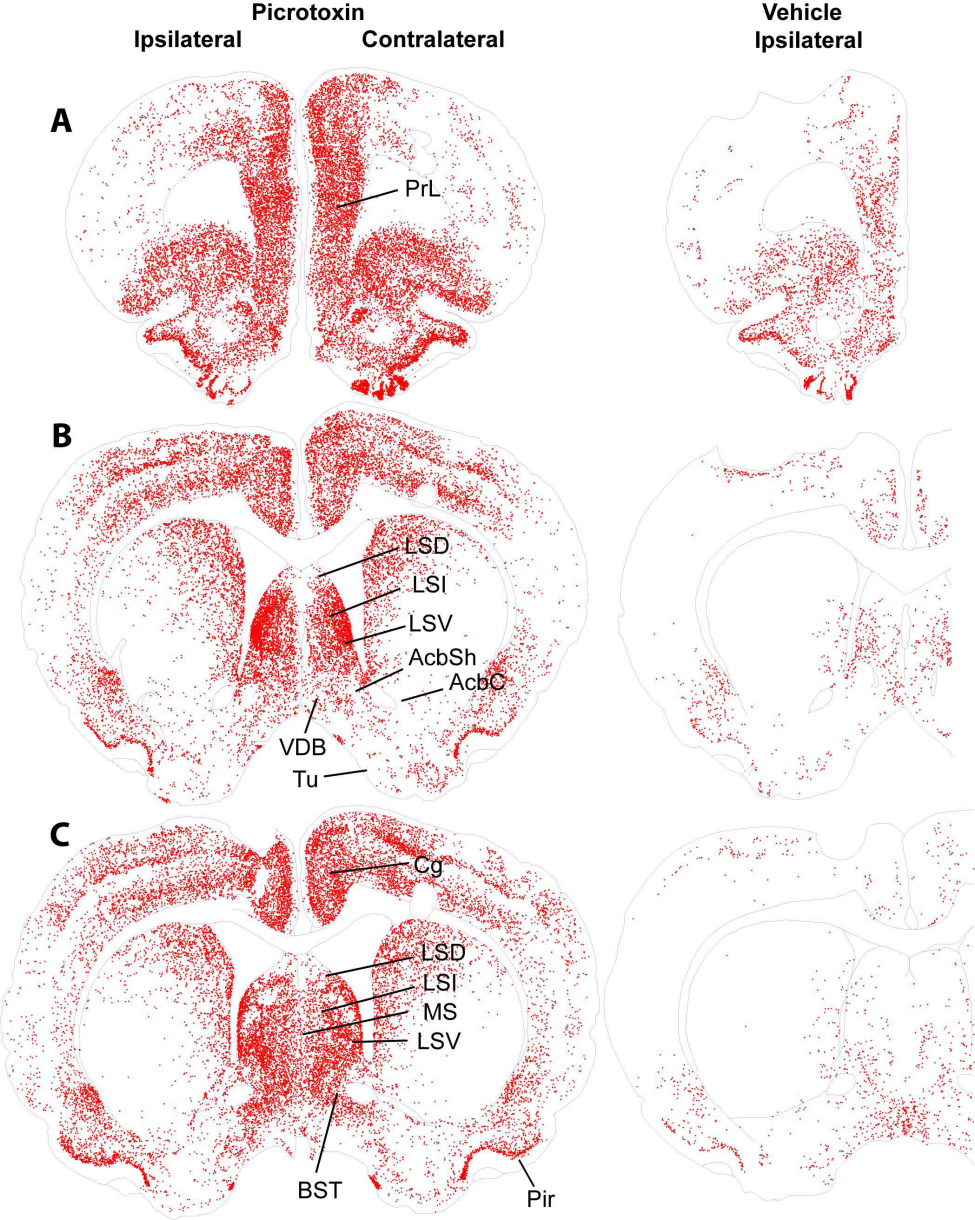
**c-Fos expression in forebrain sections of representative rats after injections of picrotoxin or vehicle into the SuM**. Each red dot indicates a nucleus containing c-Fos. Abbreviations: AcbC, nucleus accumbens core; AcbSh; nucleus accumbens shell, medial part; BST, bed nucleus of stria terminalis; Cg, cingulate cortex; LSD, lateral septal nucleus, dorsal part; LSI, lateral septal nucleus, intermediate part; LSV, lateral septal nucleus, ventral part; MS, medial septal nucleus; Pir, piriform cortex; PrL, prelimbic cortex; Tu, olfactory tubercle; VDB, nucleus of the vertical limb of the diagonal band.

**Figure 4:**
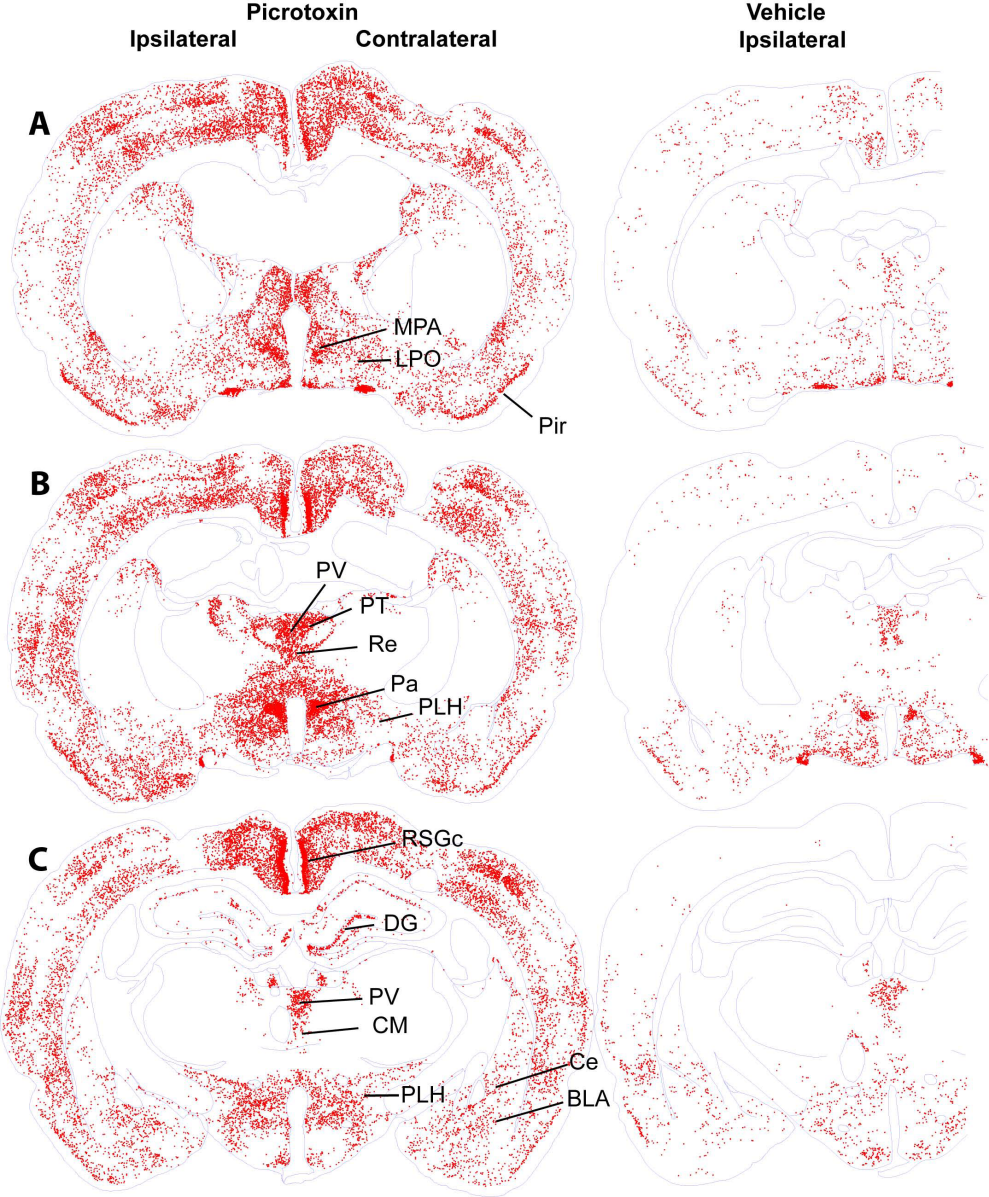
**c-Fos expression at the level of hypothalamus of representative rats after injections of picrotoxin or vehicle into the SuM**. Abbreviations: BLA, basolateral amygdaloid nucleus; Ce, central amygdaloid nucleus; CM, central medial thalamic nucleus; DG, dentate gyrus of hippocampus; LPO, lateral preoptic nucleus; MPA, medial preoptic nucleus; Pa paraventricular hypothalamic nucleus; PLH, peduncular part of lateral hypothalamus; Pir, piriform cortex; PT, paratenial thalamic nucleus; PV, paraventricular thalamic nucleus; Re, reuniens thalamic nucleus; RSGc, rostrosplenial granular cortex, c region.

**Figure 5:**
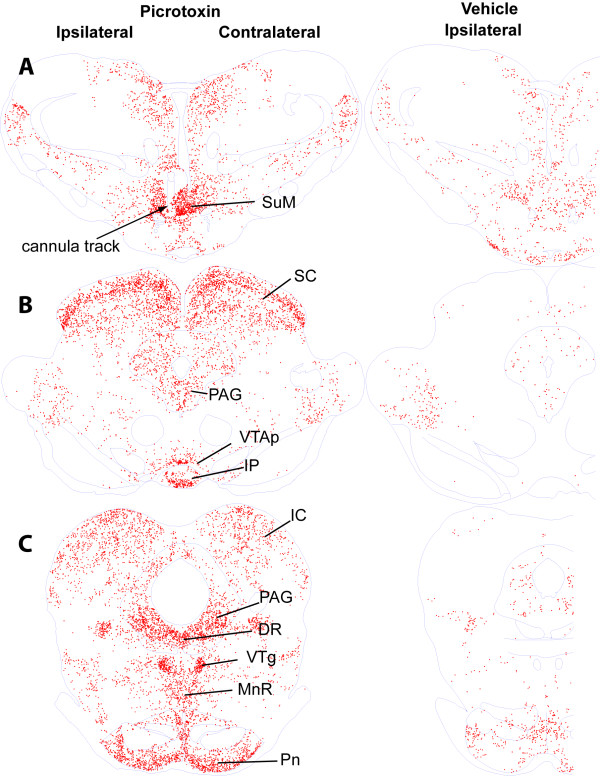
**c-Fos expression in brainstem sections of representative rats after injections of picrotoxin or vehicle into the SuM**. Abbreviations: DR, dorsal raphe nucleus; IC, inferior colliculus; IP, interpeduncular nucleus; MnR, median raphe nucleus; PAG, periaqueductal gray; Pn, pontine nuclei; SC, superior colliculus; SuM, supramammillary nucleus; VTAp, posterior ventral tegmental area; VTg, ventral tegmental nucleus of Gudden.

**Figure 6:**
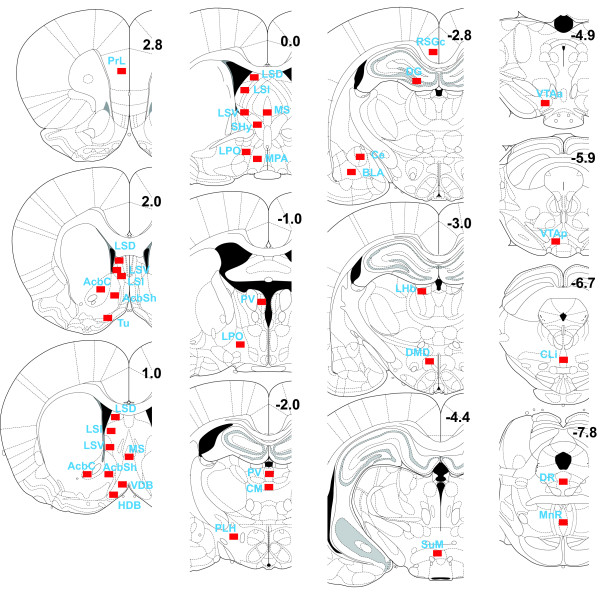
**Areas of c-Fos counts**. c-Fos expression was measured in 325 × 425 μm rectangles (shown in red) placed in respective regions of coronal sections [36]. The numbers indicate distances from bregma. Abbreviations: AcbC, nucleus accumbens core; AcbSh; nucleus accumbens shell, medial part; BLA, basolateral amygdaloid nucleus; Ce, central amygdaloid nucleus; CLi, central (caudal) linear nucleus; CM, central medial thalamic nucleus; DMD dorsomedial hypothalamic nucleus, dorsal part; DG, dentate gyrus of hippocampus; DR, dorsal raphe nucleus; HDB, nucleus of the horizontal limb of the diagonal band; LHb, lateral habenula nucleus; LPO, lateral preoptic nucleus; LSD, lateral septal nucleus, dorsal part; LSI, lateral septal nucleus, intermediate part; LSV, lateral septal nucleus, ventral part; MnR, median raphe nucleus; MPA, medial preoptic nucleus; MS, medial septal nucleus; PLH, peduncular part of lateral hypothalamus; PrL, prelimbic cortex; PV, paraventricular thalamic nucleus; RSGc, rostrosplenial granular cortex, c region; Shy, septohypothalamic nucleus; SuM, supramammillary nucleus; Tu, olfactory tubercle; VDB, nucleus of the vertical limb of the diagonal band; VTAa, anterolateral ventral tegmental area; VTAp, posterior ventral tegmental area.

**Figure 7:**
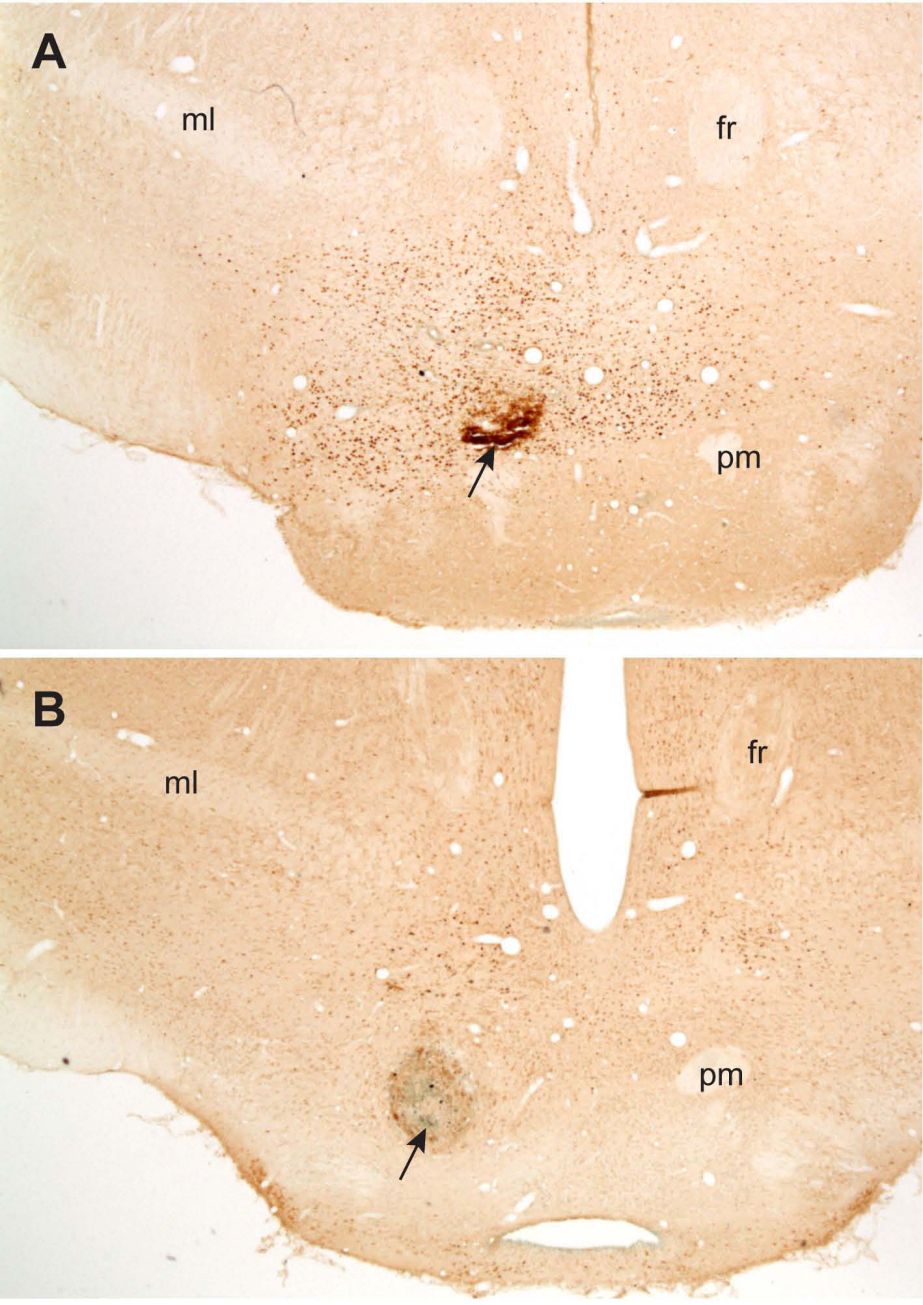
**Photomicrographs showing representative injection sites in coronal sections at the level of the supramammillary nucleus**. Arrows indicate the tips of injection cannulae for injections of picrotoxin (A) and vehicle (B). Abbreviations: fr, fascicular retroflexus; ml, medial lemniscus; pm, principal mammillary tract.

Figures 3, 4, 5 show c-Fos expression from the level of the prefrontal cortex to the level of midbrain raphe nuclei of representative rats. We also quantified c-Fos in selected brain regions (Figure 6) and summarized data in Table 1. Consistent with our hypothesis that picrotoxin administration into the SuM disinhibits its local neurons, we observed robust c-Fos expression in the SuM after picrotoxin administration into the region (Figures 5A and 7). These findings fit with our previous interpretation that self-administration and conditioned place preference induced by intra-SuM AMPA or picrotoxin are mediated by the excitation of local neurons [3,4].

**Table 1:** c-Fos counts in reward-related regions following an injection of either vehicle or picrotoxin into the SuM or its vicinity

				c-Fos counts in a 325 × 425 μm area				
				
	Region			PIC-SUM (N = 14)		PIC-OUT (N = 7)		VEH (N = 9)
				
		Distance (mm)	Hemi-sphere	Mean	P-value	Mean	P-value	Mean
Cortex	Medial prefrontal cortex	2.8	i	**71.4**	0.0042	40.7	0.2695	26.3
		
		2.8	c	**60.2**	0.0399	30.6	0.8129	27.7
	
	Retrosplenial granular cortex	-2.8	i	50.2	0.0572	**45.4**	0.0402	5.7
		
		-2.8	c	35.8		32.7		8.4
	
	Hippocampus dentate gyrus	-2.8	i	44.1		14.4		1.6
		
		-2.8	c	41.3		7.9		1.9
	
	Basolateral nucleus of amygdala	-2.8	i	11.8		18.3		3.3
		
		-2.8	c	12.9		17.3		4.6

Basal forebrain	Central nucleus of amygdala	-2.8	i	6.6		33.1		8.2
		
		-2.8	c	6.9		7.0		6.2
	
	Lateral septal nucleus, dorsal part	2.0	i	43.7	0.0565	**56.3**	0.0263	11.4
		
		2.0	c	38.7		41.1		12.2
		
		1.0	i	13.0		24.1		1.6
		
		1.0	c	10.3		22.3		1.6
		
		0.0	i	**18.4**	0.0261	6.4	0.4434	1.8
		
		0.0	c	**17.4**	0.0369	10.6	0.1427	1.3
	
	Lateral septal nucleus, intermediate part	2.0	i	**72.9**	0.0009	16.6	0.5070	5.8
		
		2.0	c	**43.9**	0.0016	13.4	0.5993	8.7
		
		1.0	i	**194.1**	0.0004	74.4	0.1148	7.7
		
		1.0	c	**99.9**	0.0045	35.4	0.3642	12.4
		
		0.0	i	**57.1**	0.0084	26.1	0.1900	4.3
		
		0.0	c	**45.3**	0.0086	17.0	0.3078	3.7
	
	Lateral septal nucleus, ventral part	2.0	i	**150.1**	0.0009	76.9	0.0730	18.0
		
		2.0	c	**86.3**	0.0084	57.0	0.0887	22.2
		
		1.0	i	**297.4**	0.0002	102.9	0.1898	32.3
		
		1.0	c	**158.1**	0.0017	52.6	0.6105	36.6
		
		0.0	i	**139.6**	0.0031	**96.9**	0.0205	8.6
		
		0.0	c	**81.1**	0.0065	**61.4**	0.0202	6.9
	
	Septohypothalamic nucleus	0.0	i	**127.1**	0.0009	55.6	0.1279	12.0
		
		0.0	c	**73.0**	0.0144	**54.9**	0.0423	11.7
	
	Medial septal nucleus	1.0	i	17.2		10.4		6.4
		
		0.0	c	**18.9**	0.0136	**14.7**	0.0290	1.7
	
	Nucleus of the vertical limb of the diagonal band	1.0	i	**21.9**	0.0356	10.0	0.2844	1.6
		
		1.0	c	6.2		10.1		1.1
	
	Nucleus of the horizontal limb of the diagonal band	1.0	i	**19.0**	0.0097	11.6	0.0778	1.6
		
		1.0	c	4.3		6.1		1.7
	
	Nucleus accumbens core	2.0	i	31.4		4.6		2.9
		
		2.0	c	**25.9**	0.0434	2.0	0.8836	3.6
		
		1.0	i	12.6		3.4		2.1
		
		1.0	c	9.6		3.4		2.8
	
	Nucleus accumbens shell, medial part	2.0	i	**48.2**	0.0012	7.1	0.8499	5.1
		
		2.0	c	**43.8**	0.0022	10.6	0.6573	6.1
		
		1.0	i	**23.4**	0.0202	12.9	0.1577	2.4
		
		1.0	c	**18.6**	0.0361	8.4	0.3962	3.4
	
	Olfactory tubercle	2.0	i	7.4		5.9		1.3
		
		2.0	c	6.4		12.0		1.9

Thalamus	Paraventricular thalamic nucleus	-1.0	i	50.2		29.4		39.2
		
		-1.0	c	41.4		29.9		36.0
		
		-2.0	m	72.1		79.4		76.1
	
	Central medial thalamic n	-2.0	m	43.6		45.4		31.1
	
	Lateral habenula	-3.0	i	18.7		18.9		18.2
		
		-3.0	c	**47.1**	0.0494	24.0	0.4813	14.7

Hypothalamus	Medial preoptic area	0.0	i	34.1		15.7		9.8
		
		0.0	c	18.6		12.1		11.3
	
	Lateral preoptic area	0.0	i	**45.5**	0.0313	**50.3**	0.0422	7.3
		
		0.0	c	30.0		37.6		8.1
		
		-1.0	i	**35.3**	0.0118	**39.9**	0.0120	2.2
		
		-1.0	c	**19.7**	0.0083	**35.9**	0.0001	2.4
	
	Dorsomedial hypothalamic nucleus	-3.0	i	**47.7**	0.0042	6.9	0.6072	12.7
		
		-3.0	c	36.6	0.0826	7.4	0.4753	15.8
	
	Lateral hypothalamic area	-2.0	i	**30.1**	0.0120	**22.0**	0.0444	4.9
		
		-2.0	c	**20.0**	0.0166	**23.1**	0.0131	4.3
	
	Supramammillary n	-4.0	m	**187.0**	0.0021	**141.0**	0.0170	48.3

Midbrain	Anterolateral VTA	-5.0	i	23.1	0.0603	4.9	0.7089	1.4
		
		-5.0	c	1.8		1.9		1.6
	
	Posterior VTA	-5.8	i	**9.6**	0.0135	4.1	0.2728	1.0
		
		-5.8	c	3.7		3.6		1.0
	
	Central linear n	-6.7	m	3.9		3.6		1.0
	
	Dorsal raphe n	-7.8	m	**21.1**	0.0251	**16.0**	0.0498	1.7
	
	Median raphe n	-7.8	m	15.4		20.1		1.3

Notes: c-Fos counts in bold indicate significant differences from respective counts for vehicle injections. Blank space in the p-value columns indicates that the effect of region was not significant, following an ANOVA/MANOVA test. Abbreviations: c, contralateral to injection site; i, ipsilateral to injection site; m, midline; n, nucleus

We observed the pattern of c-Fos expression in the VTA and the ventral striatum paralleled with the connectivity between the VTA and ventral striatum. The posterior but not anterolateral VTA had reliable increases in c-Fos expression after intra-SuM picrotoxin, while the medial nucleus accumbens shell had more reliable increases in c-Fos counts than the accumbens core (Figure 5B; Table 1). These results parallel the projection pattern of dopaminergic neurons from the VTA to the ventral striatum. The posterior VTA has been shown to predominantly project to the medial part of the ventral striatum, including the medial shell, while the anterolateral VTA largely projects to the lateral part of the ventral striatum, including the core [17]. In other words, there is mediolateral topography for dopaminergic neurons projecting from the VTA (posterior-anterolateral) to the ventral striatum (medial-lateral). This congruence between c-Fos and structure suggests that SuM picrotoxin activates the medial part of the mesolimbic dopamine system more reliably than the lateral part, and that the SuM is functionally linked with the medial mesolimbic dopamine system more closely than its' lateral counterpart. Because the medial mesolimbic dopamine system appears to mediate drugs' motivational effects more readily than the lateral portion of the system [17,18], these findings are consistent with our hypothesis that the mesolimbic dopamine system and the SuM interact for approach-related motivational processes.

In addition to the medial mesolimbic dopamine system, we observed increased expression of c-Fos in other regions that are closely connected with both the SuM and VTA. Picrotoxin administration into the SuM increased c-Fos expression in the septal complex, especially the intermediate and ventral parts of the lateral septal nucleus, the medial prefrontal cortex, the lateral preoptic area, the lateral hypothalamic area and the dorsal raphe nucleus. These regions, which are closely connected with both the SuM and VTA and expressed picrotoxin-induced c-Fos, have been previously implicated in positive motivational processes. Rats learn to self-stimulate these regions with brief electrical current [2,19-21]. Injections of drugs into these regions are rewarding, including cocaine [22] and NMDA receptor antagonists [23] in the medial prefrontal cortex, morphine and muscimol in the septal area [24-26], morphine in the lateral hypothalamic area [27,28], and the GABA_A _receptor agonist muscimol and GABA_B _receptor agonist baclofen in the dorsal raphe nucleus [29,30]. Picrotoxin administration into the SuM tended to increase, without a statistical significance, c-Fos in the medial preoptic nucleus, which is implicated in drug reward [31,32]. The same manipulation significantly increased c-Fos expression in the lateral preoptic nucleus, which supports self-stimulation [33], but has not previously been implicate in drug reward. Because the preoptic nuclei are closely connected with the SuM, VTA and the above-mentioned regions that showed significant c-Fos expression, these nuclei could play an important role in motivational processes initiated through the SuM. Thus, these findings generally support our hypothesis that these brain structures participate in motivational processes mediated through the SuM.

### c-Fos expression following brain stimulation reward

We mentioned in the background section that carbachol administration into the posterior VTA increases c-Fos expression in the SuM. Similarly, increased c-Fos expression has been found in the SuM and other brain structures that we reported here following rewarding electrical stimulation delivered at the lateral hypothalamic area [34] as well as vicinity of the dorsal raphe nucleus [35]. These previous and present findings are consistent, though not unequivocally, with the view that the SuM is a component of a network module mediating approach-type motivational processes [1].

### Limitations

To avoid interpreting the results beyond what they are, it is important to acknowledge limitations of our c-Fos technique. c-Fos expression is a correlate of manipulations and thus does not indicate any causal relationship. Accordingly, the technique cannot discriminate between reward-related neurons and other activated neurons. Thus, c-Fos expression does not necessarily mean involvement in positive motivational processes. Furthermore, c-Fos may not necessarily be expressed in every activated cell. Thus, the absence of c-Fos does not indicate the absence of neuronal activation. Finally, temporal resolution of the technique is poor. Therefore, the technique does not show how neurons were activated. In particular, our observations suggest that c-Fos expression can result from first-, second- or even third-order trans-synaptic activation. Ipsilateral sides of regions that receive monosynaptic efferents from picrotoxin-affected part of the SuM tended to have more c-Fos expression than the contralateral sides of bilateral structures, including the medial prefrontal cortex, lateral septal area, diagonal band of Broca, preoptic areas and lateral hypothalamic area (Table 1). However, c-Fos expression was generally observed bilaterally throughout the brain, including regions that do not receive efferents from the SuM (Figures 3, 4 and 5).

## Conclusion

Administration of the GABA_A _receptor antagonist picrotoxin into the SuM, which was previously found to recruit positive motivational processes related to approach/seeking, facilitated locomotor activity and induced c-Fos expression in extensive brain structures. The patterns of c-Fos expression suggest that it disinhibits local neurons and recruits activation of the mesolimbic dopamine system, prefrontal cortex, septal area, preoptic area, lateral hypothalamic area and dorsal raphe nucleus. These brain structures may be involved in approach-type motivational processes triggered by activation of supramammillary neurons.

## Methods

### Animals

Thirty male Wistar rats (Harlan, Dublin, VA, USA) weighing 250-350 g at the time of surgery were used in this experiment. They were initially housed in pairs, and after surgery, were housed individually in a humidity controlled room kept at a constant temperature and on a reverse 12-h light-dark cycle (lights on at 9:00 PM). Food and water were freely available except during testing. All procedures were approved by the Animal Care and Use Committee of the National Institute on Drug Abuse Intramural Research Program and were in accordance with the National Institutes of Health guidelines.

### Surgery

Under sodium pentobarbital (31 mg/kg, i.p.) and chloral hydrate (142 mg/kg, i.p.) anesthesia, rats were implanted unilaterally with a single guide cannula (24 gauge, Plastics One, VA, USA) into the left hemisphere that ended 1.0 mm rostral and dorsal to the supramammillary nucleus. While the injection cannula, which extended 1.0 mm beyond the tip of the guide, was not in use, a 28 gauge dummy stylet maintained the patency of the guide cannula, which extended 0.5 mm beyond the tip of the guide. Using a digital stereotaxic instrument, the following target coordinates (in millimeters) were measured: 3.8 - 4.3 posterior to bregma, 0.5 lateral to the midline, and 8.4 ventral to the skull surface. The cannulae were inserted at a 30° on the rostrocaudal plain from a rostral-to-caudal direction and a 7° on the mediolateral plain from a periphery-to-midline direction. This angular positioning of the cannulae was used to minimize tissue destruction when brains were cut for coronal sections, particularly at the level of injection site. If cannulae had been placed vertically from the dorsal entry just above the injection site, coronal sections at the level of the injection site would have been easily damaged due to the cannula track, which would have hindered c-Fos counts and our ability to locate the injection site. All depth measurements were made from the skull surface with the incisor bar set at 3.3 mm below the interaural line. The cannulae were subsequently anchored to the skull with 4 screws and dental acrylic. Animals were allowed 3 postsurgical recovery days, before we started daily handling for 2 minutes over 3 consecutive days in experimental room.

### Drugs

The GABA_A _antagonist, picrotoxin (Sigma, St. Louis, MO) was dissolved in artificial cerebrospinal fluid consisting of (in mM): 148 NaCl, 2.7 KCl, 1.2 CaCl_2_, and 0.85 MgCl_2_, pH adjusted to 6.5-7.8.

### Drug administration and behavioral monitoring

Each rat was individually placed in a Plexiglas locomotor chamber (40 × 40 × 30 cm; AccuScan Instrument, Columbus, OH) equipped with infrared detectors to record horizontal distance traveled and frequency of vertical movements (a measure of rearing incidents) for 2 hours per day for 7 consecutive days. On days 1-5, after 60 min in the locomotor chamber, each rat was allowed to become accustomed to handling procedures for microinjection without actual injections. The rat was placed in a cylinder (30 cm in diameter) for 2 min and returned to the locomotor chamber. On day 6, after the first 60 min in the locomotor chamber, each rat was placed in the cylinder, and its dummy stylet was removed and replaced with an injection cannula (31 gauge) attached to polyethylene tubing via a 10 μl Hamilton gas tight syringe controlled by a Harvard pump (model 22). The rat received vehicle (300 nl) over 60 sec, followed by additional 30 sec before the injection cannula was removed. Immediately after the dummy stylet was placed, the rat was returned to the locomotor chamber for additional 60 min. On day 7, rats received either 0.3 mM picrotoxin or vehicle with the procedure described above.

### Histology

Ninety minutes following microinjections of picrotoxin or vehicle, rats were given sodium pentobarbital (31 mg/kg, i.p.) and chloral hydrate (142 mg/kg, i.p.) anesthesia and perfused intracardially with 0.9% saline with 0.2% sodium nitrite solution followed by a 4% paraformaldehyde solution. Their brains were then removed and placed in 4% paraformaldehyde solution for 4 h before being transferred to a mixture of 18% sucrose and 2% paraformaldehye fixative for another 2 days prior to sectioning. Using a cryostat, frozen coronal sections, taken at 40 μm from the level of the prefrontal cortex to the level of the caudal pontine reticular nucleus, were submerged in phosphate buffer saline (PBS) and subsequently processed for c-Fos immunoreactivity described below. One of five consecutive sections was taken for processing. Boundaries of the SuM and other brain regions as defined by the atlas of the Paxinos and Watson [36] were determined on the basis of cytoarchitectonic features of each section. If a cannula's 0.3 mm tip was not found within the SuM, it was considered outside this structure.

### Immunohistochemical procedure

After washing in PBS, sections were incubated in 0.3% hydrogen peroxide for 10 min followed by 3 × 10 min rinses with PBS. Sections were subsequently treated with normal goat serum (Vector Laboratories, Burlingame, CA) with 0.25% Triton X-100 in PBS for 2 h. They were then incubated overnight at 4°C with anti-c-Fos rabbit antibody (1:1000, Santa Cruz Biotechnology, Santa Cruz, CA) diluted in PBS containing 0.25% Triton X-100. After rinsing 4 × 15 min in PBS, sections were incubated in a 1:200 dilution of the biotinylated secondary antibody for 2 h. After 3 × 10 min rinses with PBS, samples were treated with avidin-biotinylated enzyme complex (Vector Laboratories) for 1 h. Samples were then rinsed with PBS for 2 × 10 min and subjected to a 0.05% 3,3-diaminobenzidine-4 HCl (DAB) and 0.003% hydrogen peroxide (H_2_O_2_) solution followed by final 3 × 10 min rinses with PBS. Sections were subsequently mounted on gelatin coated slides and air-dried overnight. All sections were then incubated with Methyl Green (Vector Laboratories) for 10 min for counterstaining.

### Quantification and visualization of c-Fos

c-Fos was quantified under light microscopic examination with a 20× objective using imaging software (NIS Elements, v2.1, Nikon Instruments, Melville, NY). The sampling area was 325 × 425 μm with a 300 minimum and 3000 maximum detection threshold. We chose brain regions (Figure 6) that have been implicated in drug reward or are major projection targets of the supramammillary nucleus. c-Fos counts were recorded from brain regions of both hemispheres. In addition, we examined coronal sections of two representative brains receiving picrotoxin and vehicle for c-Fos expression throughout the brain, using a Nikon 80i light microscope equipped with a 10× objective and motorized-stage controlled by Neurolucida (MBF Bioscience, Williston, VT).

### Statistical Analyses

Data were analyzed with the ANOVA/MANOVA module of Statistica (version 6.1, StatSoft, Inc., Tulsa, OK). When the sphericity assumption examined by Mauchley Sphericity Test was violated for repeated factors, effects of the repeated factors were analyzed by MANOVAs; otherwise ANOVAs were used. When factors with more than two levels were found to be significant, we performed Newman-Keuls posthoc tests. Pearson's product-moment correlation coefficients were calculated between c-Fos counts and locomotion or rearing scores (during the first 30 min) among all the rats including rats received picrotoxin outside of the SuM and vehicle (N = 30).

## List of Abbreviations

PBS: phosphate buffer solution; SuM: supramammillary nucleus; VTA: ventral tegmental area.

## Authors' contributions

SI conceived the study; RS and SI designed experiments; RS conducted experiments and measured c-Fos expression; RS and SI analyzed data and wrote the paper. Both authors read and approved the final manuscript.
